# Use of Quantitative Morphological and Functional Features for Assessment of Axillary Lymph Node in Breast Dynamic Contrast-Enhanced Magnetic Resonance Imaging

**DOI:** 10.1155/2018/2610801

**Published:** 2018-05-30

**Authors:** Roberta Fusco, Mario Sansone, Vincenza Granata, Maurizio Di Bonito, Franca Avino, Orlando Catalano, Gerardo Botti, Antonella Petrillo

**Affiliations:** ^1^Radiology Unit, “Dipartimento di Supporto ai Percorsi Oncologici Area Diagnostica, Istituto Nazionale Tumori-IRCCS-Fondazione G. Pascale”, Via Mariano Semmola, Naples, Italy; ^2^Department of Electrical Engineering and Information Technologies, University “Federico II” of Naples, Via Claudio, Naples, Italy; ^3^Pathology Unit, “Dipartimento di Supporto ai Percorsi Oncologici Area Diagnostica, Istituto Nazionale Tumori-IRCCS-Fondazione G. Pascale”, Via Mariano Semmola, Naples, Italy; ^4^Senology Surgery Unit, “Dipartimento Corp-S Assistenziale e di Ricerca dei Percorsi Oncologici del Distretto Toracico, Istituto Nazionale Tumori-IRCCS-Fondazione G. Pascale”, Via Mariano Semmola, Naples, Italy

## Abstract

**Background:**

Axillary lymph-node assessment is considered one of the most important prognostic factors concerning breast cancer survival.

**Objective:**

We investigated the discriminative power of morphological and functional features in assessing the axillary lymph node.

**Methods:**

We retrospectively analysed data from 52 consecutive patients who undergone DCE-MRI and were diagnosed with primary breast carcinoma: 94 lymph nodes were identified. Per each lymph node, we extracted morphological features: circularity, compactness, convexity, curvature, elongation, diameter, eccentricity, irregularity, radial length, entropy, rectangularity, roughness, smoothness, sphericity, spiculation, surface, and volume. Moreover, we extracted functional features: time to peak (TTP), maximum signal difference (MSD), wash-in intercept (WII), wash-out intercept (WOI), wash-in slope (WIS), wash-out slope (WOS), area under gadolinium curve (AUGC), area under wash-in (AUWI), and area under wash-out (AUWO). Selection of important features in predicting metastasis has been done by means of receiver operating characteristic (ROC) analysis. Performance of linear discriminant analysis was analysed.

**Results:**

All morphological features but circularity showed a significant difference between median values of metastatic lymph nodes group and nonmetastatic lymph nodes group. All dynamic parameters except for MSD and WOS showed a statistically significant difference between median values of metastatic lymph nodes group and nonmetastatic lymph nodes group. Best results for discrimination of metastatic and nonmetastatic lymph nodes were obtained by AUGC (accuracy 75.8%), WIS (accuracy 71.0%), WOS (accuracy 71.0%), and AUCWO (accuracy 72.6%) for dynamic features and by compactness (accuracy 82.3%), curvature (accuracy 71.0%), radial length (accuracy 71.0%), roughness (accuracy 74.2%), smoothness (accuracy 77.2%), and speculation (accuracy 72.6%) for morphological features. Linear combination of all morphological and/or of all dynamic features did not increase accuracy in metastatic lymph nodes discrimination.

**Conclusions:**

Compactness as morphological feature and area under time-intensity curve as dynamic feature were the best parameters in identifying metastatic lymph nodes on breast MRI.

## 1. Background 

In 2017, breast cancer had the highest incidence among female cancers and is still the second (after lung) leading cause of death from cancer in the US [1]. The transition from nonmetastatic to metastatic state of breast cancer is characterised by the diffusion of the primary lesion towards lymphatic sites. Therefore, accurate evaluation of metastasis in axillaries lymphatic nodes is a crucial factor affecting medical management, surgery, and prognosis [2–4].

Sentinel lymph-node biopsy (SLNB) has been effectively used for identifying, via radiotracer and/or blue dye, the nodes draining the breast which are possibly the first to be encountered during tumor spreading [5]. SLNB is commonly executed after surgical removal of the primary lesion and has shown an accuracy of 93.5 to 97.5% [6, 7]. However, it has been noticed that SLNB can have long-term morbidity that potentially can affect the quality of life despite being less significant than axillary lymph-node dissection [8, 9]. In addition, preoperative evaluation of axillary lymph nodes might improve patient-based treatment: in fact, options might include neoadjuvant chemotherapy, intraoperative breast radiotherapy, and reconstruction planning. Moreover, when metastatic axillary disease is diagnosed before surgery, the surgeon can discuss specific aspects of axillary lymph-node dissection with the patient.

Despite being not very accurate, imaging techniques such as ultrasound (US), computed tomography (CT), and positron emission tomography (PET)/CT are often used in clinical practice [10–13]. Breast magnetic resonance imaging (MRI), because of its versatility, has gained a large consensus over the past two decades and many technological improvements have contributed to its diffusion [12]. In particular, dynamic contrast-enhanced magnetic resonance imaging (DCE-MRI) has been shown to be able to distinguish benign from malignant breast lesions by means of simultaneous evaluation of morphological and functional information. At the time of writing, axillary lymph nodes evaluation via DCE-MRI has not yet been introduced in clinical practice. Mainly, diagnostic criteria for malignancy of axillary lymph nodes are based exclusively on morphology; however, these are still controversial [11, 14, 15].

In this study, we investigated the discriminative power of MRI in both morphological and functional features derived by dynamic contrast-enhanced MRI (DCE-MRI) for axillary lymph-node evaluation. We attempted to identify the best quantitative feature to discriminate metastatic from nonmetastatic lymph nodes among 26 morphological and functional parameters and their linear combinations.

## 2. Methods

### 2.1. Patients Inclusion Criteria

A prospectively collected database has been reviewed after Institutional Review Board approval. We identified 268 consecutive patients from February 2009 to December 2013 for newly diagnosed breast carcinoma. All these subjects had undergone DCE-MRI in a single cancer centre. The study population comprised 52 patients with breast cancer who also underwent pathological evaluation of axillary lymph nodes. Age ranged from 31 to 58 years. Patients included in the study (1) had breast cancer with clinical evaluation (TNM score) T1-T2 and (2) underwent SLNB or/and axillary lymphadenectomy. Patients who carried an implanted device, were pregnant, or had any contraindication for MRI were not included in the study. In addition, we excluded patients having undergone radiation therapy or chemotherapy within 12 months before the MRI. All patients provided informed consent to the use of their data for research purposes. This retrospective study was performed according to regulations issued by our local Institutional Review Board.

### 2.2. MRI Methodology

DCE-MRI has been executed using 1.5 T breast-dedicated equipment (Aurora; Aurora Imaging Technology, North Andover, USA), embodying an in-table coil [14]. Exams were arranged from the 7th to 14th day of the menstrual cycle in premenopausal women; no scheduling limitations were applied in postmenopausal women.

The sequence used for precontrast imaging was a three-dimensional (3D) nonspoiled SPIRAL-RODEO fat-sat (TR 29 ms, TE 4.8 ms, flip angle 45°, matrix 512 × 512, thickness 1.13 mm, and gap 1.13 mm); after contrast injection, four dynamic 3D spoiled SPIRAL-RODEO fat-sat acquisitions (TR 29 ms, TE 4.8 ms, flip angle 45°, matrix 512 × 512, thickness 1.13 mm, and gap 1.13 mm) were used. The time interval between acquisitions was 90 s. A bolus of gadobenate dimeglumine (Multihance, Gd-BOPTA Bracco; Atlanta Pharma, Konstanz, Germany) has been intravenously injected using a dose of 0.1 mmol/kg body weight at a flow rate of 2 ml/s, followed by 20 ml of saline solution at the same rate. An automatic contrast delivery system was employed (Optistar Elite, Covidien Imaging Solution, Hazelwood, USA).

### 2.3. Histopathological Evaluation and Operation of Axillary Lymph Nodes

Samples of SLNB were assessed by immediate frozen section and hematoxylin and eosin staining. The nodes were subsequently submitted for permanent sectioning and immunohistochemical assay. According to the American Joint Committee on Cancer guidelines for breast cancer staging [16], a patient with isolated tumor cells was considered node-negative and did not undergo any additional lymph-node surgery.

### 2.4. Images Analysis

**Figure 1:**
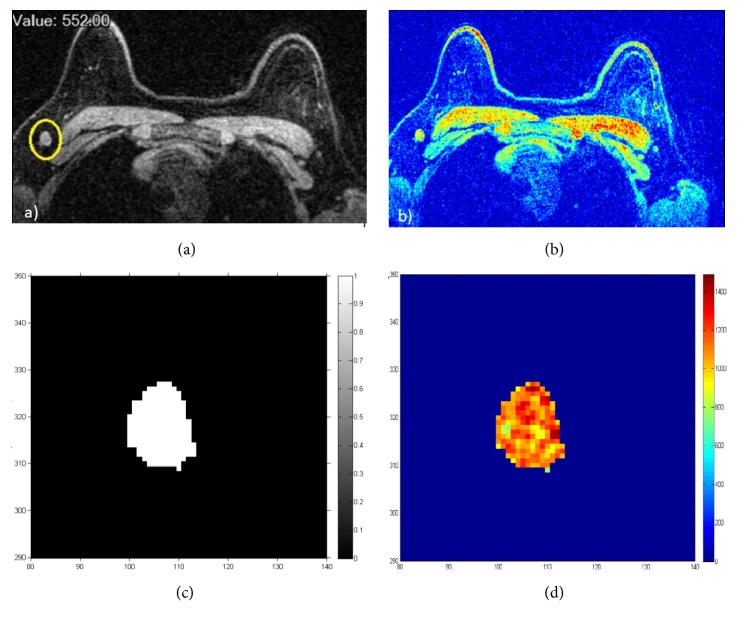
Lymph-node illustration on contrast-enhanced MR imaging and their segmentation for a single slice: (a)–(c) grey level; (b)–(d) RGB values.

Two radiologists having more than 15 years of experience (AP) and more than 10 years of experience (SF), respectively, reviewed images. For each lymph node having a lower diameter ≥ 10 mm, the manual segmentation was made using OsiriX v.3.8.1, on the data acquired after contrast injection using a pulse sequence for three-dimensional fat-saturated axial nonspoiled SPIRAL-RODEO images (Figure 1). Per each lymph node, on each slice, a region of interest (ROI) was drawn: the set of all ROIs corresponding to a single lymph node formed a Volume of Interest (VOI). ROI border has been placed in the lymph-node periphery close to the margin. Lymph nodes were evaluated using quantitative descriptors involving morphological and dynamic parameters.

### 2.5. Dynamic Parameters

**Figure 2:**
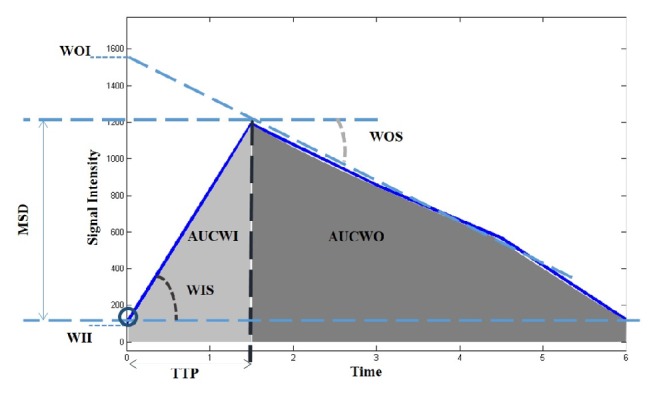
Dynamic parameters illustration.

Nine dynamic features emerged from the literature [17–20], which were extracted using the approach previously reported in a previous publication from our group [19] (Figure 2): maximum signal difference (MSD), the time to peak (TTP) between the wash-in (WI) and wash-out (WO) segments, the WI slope (WIS), the WO slope (WOS), the WI intercept (WII), the WO intercept (WOI), the area under curve (AUC), the area under WI tract (AUCWI), and the area under WO tract (AUCWO).

### 2.6. Feature Extraction

Per each VOI, 17 morphological features were calculated [21–23]. Before feature computation, all lymph-node binary masks have been reinterpolated on a common grid of equal size (1 × 1 × 1 mm^3^) in three orthogonal directions. A brief description of all morphological features has been provided in Table 1. Detailed mathematical definitions might slightly vary among studies; therefore, we report the specific definitions we used:

1. Circularity = (volume of the sphere with average lymph-node radius)/(lymph-node volume).
2. Compactness = (lymph-node surface area)/(lymph-node volume).
3. Irregularity = 1 – (surface area of the sphere with average lymph-node radius)/(lymph-node surface area).
4. Diameter = diameter of the sphere corresponding to the lymph-node volume.
5. Rectangularity = (lymph-node volume)/(volume of the smallest parallelepiped containing the lymph node).
6. Radial length = average distance between boundary points and lymph-node barycentre.
7. Volume = number of voxels of the lymph node times the volume of a single voxel.
8. Smoothness = (1/*N*)∑_*n*_*R*_*n*_ − (*R*_*n*−1_ + *R*_*n*+1_)/2, where *Rn* is the *n*th boundary point distance from the barycentre along a lymph-node slice.
9. Curvature = average(abs(*x*′*y*′′–*y*′*x*′′)/(*x*^′2^ +* y*^′2^)^(3/2)^), where *x*,*y* are the coordinate parametric representation of the boundary points along a lymph-node slice,* x*′*y*′ are the first derivative with respect to the parameter, and* x*′′*y*′′ are the second derivative.
10. Roughness: ([(1/*N*)∑_*n*=1_^*N*^(*R*_*n*_ − *μ*)^4^]^1/4^ − [(1/*N*)∑_*n*=1_^*N*^(*R*_*n*_ − *μ*)^2^]^1/2^)/*μ*, where *N* is the number of points of the boundary, *Rn* is the radial distance of the *n*th point, and *μ* is the average radial distance.
11. Sphericity = (average radial length)/(standard deviation radial length).
12. Eccentricity = (lymph-node largest diameter)/(the largest diameter orthogonal to the previous one).
13. Surface = number of voxels belonging to the lymph-node boundary.
14. Spiculation = standard deviation of radial length.
15. Convexity = (convex-hull volume)/(lymph-node volume);
16. Entropy: −∑_*n*_*P*(*R*_*n*_ = *r*_*n*_)log⁡*P*(*R*_*n*_ = *r*_*n*_), where *P*(*R* = *rn*) is the distribution of radial length.
17. Elongation = (length)/(width) of the smallest rectangle containing the lymph node averaged per each slice in three orthogonal directions.

### 2.7. Statistical Analysis

Histopathological results after surgical intervention served as a reference standard for *N* staging. For each parameter, median and standard deviation (SD) were calculated as representative values of segmented VOI. Interobserver agreement was calculated to assess the variability between two readers in the manual lymph-nodes segmentation. As is commonly reported, an interobserver correlation coefficient of 0–0.20 reflected a poor agreement, of 0.21–0.40 reflected a fair agreement, of 0.41–0.60 reflected a moderate agreement, of 0.61–0.80 reflected a good agreement, and of 0.81–1.00 reflected an excellent agreement. The nonparametric Mann–Whitney test was used to emphasize statistically significant difference between median values of morphological and dynamic parameters in metastatic lymph-nodes group versus nonmetastatic lymph-nodes group. A *P* value of <0.05 was considered significant for all tests.

Receiver operating characteristic (ROC) analysis in addition to sensitivity, specificity, misclassification error (number of false negatives and false positives over the total), and accuracy (number of true negatives and true positives over the total) has been performed with respect to histopathological results.

Moreover, we applied a linear discriminant analysis (LDA) [24] to identify the best weighted linear combination of features producing the best results considering, respectively, morphological features only, dynamic features only, and both kinds of features together (sensibility and specificity were reported and were considered significant for the features with an accuracy of >70% at ROC analysis). 10-fold cross-validation has been performed in order to have robust result [24].

Statistical processing and classification have been performed by means of the Statistics Toolbox within Matlab R2007a (MathWorks Inc., Natick, USA).

## 3. Results

In the present study, 94 dominant lymph nodes were evaluated in 52 patients with primary breast carcinoma: 48 metastatic lymph nodes and 46 not pathological lymph nodes.

Table 2 reports median and standard deviation for each morphological parameter in the metastatic lymph-nodes group versus the nonmetastatic lymph-nodes group. The median of all the parameters, except circularity, showed a statistically significant difference between the two groups.

Table 3 reports median and standard deviation for each dynamic parameter in the metastatic lymph-nodes group versus the nonmetastatic lymph-nodes group. The median of all parameters, except MSD and WOS, showed a statistically significant difference between the two groups.

Interobserver correlation coefficient calculated on VOI for each segmented lymph node was of 0.864 (95% CI: 0.835–0.884) indicating an excellent agreement between the two manual segmentations; in addition, this indicates the robustness of morphological and dynamic parameters calculated on segmented lymph nodes.

Table 4 reports findings of ROC analysis for each morphological and dynamic parameter in terms of sensitivity, specificity, misclassification error, and accuracy. The best discrimination between metastatic lymph nodes and nonmetastatic lymph nodes has been obtained by AUC, WIS, WOS, and AUCWO of the dynamic features and by compactness, curvature, radial length, roughness, smoothness, and speculation of the morphological features.

Table 5 reports the finding of LDA analysis when all morphological and dynamic features were considered and when the linear combination of significant morphological and dynamic features was considered.

## 4. Discussion

Histopathologic staging of axillary lymph node is one of the most commonly used predictors of breast cancer survival. Currently, diagnosis of metastatic involvement requires invasive procedures such as pathologic assessment of biopsy tissue or postsurgery dissection. Conventional MRI with double breast coils can noninvasively evaluate both breasts and simultaneously assess axillary lymph nodes; moreover, new techniques, such as DCE-MRI, are now achieving a sufficient degree of maturity for breast cancer evaluation. The verification of MRI-based diagnoses of a specific node with histopathologic analysis of the same node is still a challenge. Moreover, the use of DCE-MRI for assessment of metastatic axillary lymph nodes has not yet been sufficiently investigated and conflicting results until now have been published [25–32]. In this study, we used several morphological features and several dynamic MRI characteristics of axillary lymph nodes. We investigated whether and how malignant nodes could be assessed preoperatively and noninvasively by means of MRI using both morphologic and dynamic criteria. The sensitivity of these features ranged from 28.6% to 92.9%, and the specificity ranged from 34.6% to 81.3%. The best results for discrimination of nonmetastatic lymph nodes by metastatic lymph nodes have been achieved by AUC, WIS, WOS, and AUCWO, among the dynamic features, and by compactness, curvature, radial length, roughness, smoothness, and speculation, among morphological features. The best dynamic parameter was AUC reporting a sensitivity, specificity, misclassification error, and accuracy of 81%, 72%, 24%, and 76%, respectively. The best morphological parameter was compactness reporting a sensitivity, specificity, misclassification error, and accuracy of 83%, 81%, 18%, and 82%, respectively.

Our results are similar to those of other researchers [25–29]. Choi et al. [25] performed a meta-analysis reporting the diagnostic performance of CT, MRI, and PET/CT for detection of metastatic lymph nodes in cervical cancer patients: for region- and node-based data analysis, MRI sensitivity and specificity were 38% and 97%, respectively. He et al. [26] and Baltzer et al. [27] investigated diagnostic performance of specific morphological and/or dynamic features obtained by MR imaging. He et al. reported the area under ROC (AUROC) for short and long lymph-node axis of 0.89 and 0.74, respectively (sensitivity of 93.3% and specificity of 72.6% for short axis, sensitivity of 88.1% and specificity of 64.1% for long axis) and the AUROC of early stage enhancement rate as a dynamic feature (sensitivity of 97.0% and specificity of 73.5%). Baltzer et al. [27] investigated only the margin of lymph nodes as a morphological parameter reporting a sensitivity of 41.2% and a specificity of 95.2%. Schacht et al. [28] reported the results of a quantitative breast MR image analysis for classification of axillary lymph nodes. The best features in that study were the circularity as a morphological parameter with AUROC of 0.67 and the wash-out rate with AUROC of 0.62. Harada et al. [29] evaluated the diagnostic performance of morphologic features computable from MR images using a contrast agent actually not commercialized (ultrasmall superparamagnetic iron oxide): sensitivity, specificity, and overall accuracy were 36.5%, 94.1%, and 81%, respectively.

On the basis of our results, a linear combination of morphological and dynamic feature does not increase the accuracy in lymph-nodes discrimination. LDA results of each group and combination of groups (morphological and/or dynamic parameters) were comparable to results of dynamic and morphological parameters considered separately. A future endpoint could be to perform multivariate analysis of functional parameters including other modalities such as PET/CT examination or quantitative parameters derived by hybrid system like PET/MRI [33].

A limit of our study consists of the manual segmentation of lymph nodes. However, an expert breast radiologist performed this procedure. A second limitation is the level of complexity in the “gold standard” due to the difficult task of identifying which lymph nodes were biopsied or dissected and of matching the pathologic results to the imaged nodes.

## Figures and Tables

**Table 1:** List of features used in analysis with definitions.

Feature category	Feature	Description
Dynamic	TTP	Time to peak
	MSD	Maximum signal difference
	AUGC	Area under gadolinium curve
	AUCWI	Area under gadolinium curve in the wash-in phase
	AUCWO	Area under gadolinium curve in the wash-out phase
	WIS	Wash-in slope
	WII	Wash-in intercept
	WOS	Wash-out slope
	WOI	Wash-out intercept

Morphological	circularity	Similarity of the lesion shape to a sphere
	compactness	Ratio between surface and volume
	convexity	Ratio between the smallest volume with convex curvature that contains the lymph node and its volume
	curvature	Measure of curvature of lymph node contour
	elongation	Parameter that estimates how much the ROI is pronounced along one direction than along the other
	diameter	Diameter of the sphere having the same ROI volume
	eccentricity	Ratio of the larger rope and the largest among the orthogonal ropes
	irregularity	Deviation of the lesion surface from the surface of a sphere
	radial length	Average distance between points on the border and the center of the lymph node
	entropy	Entropy of radial length
	rectangularity	Similarity of the lesion shape to a rectangle
	roughness	Distances of each point of the center than the radial length average
	smoothness	Measurement of lymph node contour irregularities
	sphericity	Ratio between the average radial length and the standard deviation of the rays
	spiculation	Standard deviation of the radial lengths with respect to the radial length average
	surface	Sum of lymph nodes contour pixels
	volume	Volume of the entire lymph node

**Table 2:** Morphological features median and standard deviation values for each parameter. *P* value was calculated using Mann–Whitney test.

Morphological Features	Metastatic		Non Metastatic		*P* value
	Lymph-nodes		Lymph-nodes		
	Median	SD	Median	SD	
circularity	0,001	0,002	0,002	0,003	0,829
compactness	0,339	0,125	0,453	0,076	0,021
Convexity	0,824	0,133	0,857	0,159	0,005
curvature	0,091	0,038	0,133	0,052	0,001
Elongation	1,133	0,307	1,281	0,430	0,000
diameter	17,812	7,891	14,392	3,278	0,000
eccentricity	1,658	0,584	1,679	0,678	0,000
Irregularity	−3,487	0,562	−3,425	0,760	0,000
radial length	0,929	0,031	0,960	0,020	0,000
Entropy	4,763	0,214	4,664	0,241	0,000
rectangularity	0,358	0,071	0,332	0,092	0,000
Roughness	0,353	0,232	0,102	0,078	0,000
smoothness	4,283	1,914	3,320	0,539	0,000
sphericity/roundness	26,372	15,870	41,890	27,259	0,000
Spiculation	6,602	2,656	4,323	1,369	0,000
Surface	1094,000	1207,675	698,000	540,839	0,000
volume	2959,000	7956,682	1561,000	1114,509	0,000

**Table 3:** Dynamic features median and standard deviation values for each parameter. *P* value was calculated using Mann–Whitney test.

Features	Metastatic Lymph-nodes		Non Metastatic Lymph-nodes		*P* value
	Median	SD	Median	SD	
MSD	1079,000	641,773	597,500	474,324	1,000
TTP	372,261	169,279	459,888	179,981	0,021
AUC	3,000	1,193	3,000	0,994	0,003
WII	1,453	0,215	1,521	0,198	0,001
WOI	11475,000	3002,216	8871,375	2198,225	0,000
WIS	1924,637	819,422	2005,110	668,567	0,037
WOS	1384,000	535,408	1442,450	486,190	0,214
AUCWI	395,952	174,820	468,695	179,586	0,002
AUCWO	2951,250	1549,637	3654,000	1280,761	0,000

**Table 4:** ROC analysis findings for each morphological and dynamic parameter in terms of sensitivity, specificity, misclassification error, and accuracy.

Features	Sensitivity [%]	Specificity [%]	Misclassification Error [%]	Accuracy [%]	AUC
MSD	73,910	64,100	32,260	67,740	0.668
TTP	38,890	34,620	62,900	37,100	0.516
AUC	80,770	72,220	24,190	*75,810*	*0.769*
WII	40,000	43,240	58,060	41,940	0.501
WOI	44,830	45,450	54,840	45,160	0.437
WIS	78,260	66,670	29,030	*70,970*	*0.717*
WOS	70,970	70,970	29,030	*70,970*	*0.657*
AUCWI	28,570	39,020	64,520	35,480	0.459
AUCWO	81,820	67,500	27,420	*72,580*	*0.766*
Circularity	60,980	71,430	35,480	64,520	0.762
Compactness	83,330	81,250	17,740	*82,260*	*0.824*
Convexity	42,420	41,380	58,060	41,940	0.469
Curvature	68,570	74,070	29,030	*70,970*	*0.770*
Elongation	57,890	62,500	40,320	59,680	0.680
Diameter	69,230	63,890	33,870	66,130	0.738
Eccentricity	52,630	54,170	46,770	53,230	0.453
Irregularity	54,550	55,170	45,160	54,840	0.448
Radial Length	76,000	67,570	29,030	*70,970*	*0.811*
Entropy	62,500	72,730	33,870	66,130	0.699
Rectangularity	56,670	56,250	43,550	56,450	0.564
Roughness	85,710	68,290	25,810	*74,190*	*0.834*
Smoothness	90,480	70,730	22,580	*77,420*	*0.810*
Sphericity/roundness	62,790	78,950	32,260	67,740	0.710
Spiculation	81,820	67,500	27,420	*72,580*	*0.742*
Surface	68,180	60,000	37,100	62,900	0.713
Volume	92,860	62,500	30,650	69,350	0.738

**Table 5:** LDA analysis findings when all morphological and dynamic features were considered and when the linear combinations of significant morphological and dynamic features were considered.

	Sensitivity [%]	Specificity [%]	AUC
All dynamic features	77,420	70,970	0.778
All morphological features	70,970	80,650	0.803
All features	64,520	77,400	0.754
All significant dynamic features	85,000	66,700	0.794
All significant morphologic features	88,500	77,800	0.812
All significant features	81,000	65,900	0.789

## References

[1] https://www.cancer.org/content/dam/cancer-org/research/cancer-facts-and-statistics/annual-cancer-facts-and-figures/2017/cancer-facts-and-figures-2017.pdf

[2] Le Bouedec G., Gauthier T., Gimbergues P., Dauplat J. (2008). Axillary recurrence after negative sentinel lymph node biopsy in breast cancer. *La Presse Médicale*.

[3] Strnad P., Rob L., Krízová H., Zuntová A., Chod J., Halaska M. (2005). Sentinel lymphatic node biopsy for breast cancer in practice. *Česká Gynekologie*.

[4] Beenken S. W., Urist M. M., Zhang Y. (2003). Axillary Lymph Node Status, but Not Tumor Size, Predicts Locoregional Recurrence and Overall Survival after Mastectomy for Breast Cancer. *Annals of Surgery*.

[5] McLaughlin S. A., Wright M. J., Morris K. T. (2008). Prevalence of lymphedema in women with breast cancer 5 years after sentinel lymph node biopsy or axillary dissection: Objective measurements. *Journal of Clinical Oncology*.

[6] Veronesi U., Paganelli G., Viale G. (2003). A randomized comparison of sentinel-node biopsy with routine axillary dissection in breast cancer. *The New England Journal of Medicine*.

[7] Krag D. N., Anderson S. J., Julian T. B. (2007). Technical outcomes of sentinel-lymph-node resection and conventional axillary-lymph-node dissection in patients with clinically node-negative breast cancer: results from the NSABP B-32 randomised phase III trial. *The Lancet Oncology*.

[8] Crane-Okada R., Wascher R. A., Elashoff D., Giuliano A. E. (2008). Long-term morbidity of sentinel node biopsy versus complete axillary dissection for unilateral breast cancer. *Annals of Surgical Oncology*.

[9] Purushotham A. D., Upponi S., Klevesath M. B. (2005). Morbidity after sentinel lymph node biopsy in primary breast cancer: results from a randomized controlled trial. *Journal of Clinical Oncology*.

[10] Vassallo P., Edel G., Roos N., Naguib A., Peters P. E. (1993). In-vitro high-resolution ultrasonography of benign and malignant lymph nodes: A sonographic-pathologic correlation. *Investigative Radiology*.

[11] Dooms G. C., Hricak H., Crooks L. E., Higgins C. B. (1984). Magnetic resonance imaging of the lymph nodes: Comparison with CT. *Radiology*.

[12] Ueda S., Tsuda H., Asakawa H. (2008). Utility of 18F-fluoro-deoxyglucose emission tomography/ computed tomography fusion imaging (18F-FDG PET/CT) in combination with ultrasonography for axillary staging in primary breast cancer. *BMC Cancer*.

[13] Cooper K. L., Meng Y., Harnan S. (2011). Positron emission tomography (PET) and magnetic resonance imaging (MRI) for the assessment of axillary lymph node metastases in early breast cancer: systematic review and economic evaluation.. *Health Technology Assessment*.

[14] Memarsadeghi M., Riedl C. C., Kaneider A. (2006). Axillary lymph node metastases in patients with breast carcinomas: assessment with nonenhanced versus USPIO-enhanced MR imaging. *Radiology*.

[15] Luciani A., Pigneur F., Ghozali F. (2009). Ex vivo MRI of axillary lymph nodes in breast cancer. *European Journal of Radiology*.

[16] Edge S. B., Byrd D. R., Compton C. C., Fritz A. G., Greene F. L., Trotti A. (2010). *American Joint Commision on Cancer. Breast Cancer: Prespectives on Anatomic Staging—Based on The AJCC Staging Manual*.

[17] Fusco R., Sansone M., Filice S. (2015). Integration of DCE-MRI and DW-MRI Quantitative Parameters for Breast Lesion Classification. *BioMed Research International*.

[18] Schlossbauer T., Leinsinger G., Wismuller A. (2008). Classification of small contrast enhancing breast lesions in dynamic magnetic resonance imaging using a combination of morphological criteria and dynamic analysis based on unsupervised vector-quantization. *Investigative Radiology*.

[19] Fusco R., Petrillo A., Petrillo M., Sansone M. (2013). Use of tracer kinetic models for selection of semi-quantitative features for DCE-MRI data classification. *Applied Magnetic Resonance*.

[20] Krzanowski W. J. (1988). *Principles of multivariate analysis*.

[21] Ikeda D. M., Hylton N. M., Kinkel K. (2001). Development, standardization, and testing of a lexicon for reporting contrast-enhanced breast magnetic resonance imaging studies. *Journal of Magnetic Resonance Imaging*.

[22] McLaren C. E., Chen W.-P., Nie K., Su M.-Y. (2009). Prediction of Malignant Breast Lesions from MRI Features. A Comparison of Artificial Neural Network and Logistic Regression Techniques. *Academic Radiology*.

[23] Sansone M., Fusco R., Petrillo A., Petrillo M., Bracale M. (2011). An expectation-maximisation approach for simultaneous pixel classification and tracer kinetic modelling in dynamic contrast enhanced-magnetic resonance imaging. *Medical & Biological Engineering & Computing*.

[24] Fusco R., Sansone M., Filice S. (2016). Pattern Recognition Approaches for Breast Cancer DCE-MRI Classification: A Systematic Review. *Journal of Medical and Biological Engineering*.

[25] Choi H. J., Ju W., Myung S. K., Kim Y. (2010). Diagnostic performance of computer tomography, magnetic resonance imaging, and positron emission tomography or positron emission tomography/computer tomography for detection of metastatic lymph nodes in patients with cervical cancer: Meta-analysis. *Cancer Science*.

[26] He N., Xie C., Wei W. (2012). A new, preoperative, MRI-based scoring system for diagnosing malignant axillary lymph nodes in women evaluated for breast cancer. *European Journal of Radiology*.

[27] Baltzer P. A. T., Dietzel M., Burmeister H. P. (2011). Application of MR mammography beyond local staging: Is there a potential to accurately assess axillary lymph nodes? Evaluation of an extended protocol in an initial prospective study. *American Journal of Roentgenology*.

[28] Schacht D. V., Drukker K., Pak I., Abe H., Giger M. L. (2015). Using quantitative image analysis to classify axillary lymph nodes on breast MRI: A new application for the Z 0011 Era. *European Journal of Radiology*.

[29] Harada T., Tanigawa N., Matsuki M., Nohara T., Narabayashi I. (2007). Evaluation of lymph node metastases of breast cancer using ultrasmall superparamagnetic iron oxide-enhanced magnetic resonance imaging. *European Journal of Radiology*.

[30] Luciani A., Dao T. H., Lapeyre M. (2004). Simultaneous Bilateral Breast and High-Resolution Axillary MRI of Patients with Breast Cancer: Preliminary Results. *American Journal of Roentgenology*.

[31] Hwang S. O., Lee S.-W., Kim H. J., Kim W. W., Park H. Y., Jung J. H. (2013). The comparative study of ultrasonography, contrast-enhanced MRI, and 18F-FDG PET/CT for detecting axillary lymph node metastasis in T1 breast cancer. *Journal of Breast Cancer*.

[32] Valente S. A., Levine G. M., Silverstein M. J. (2012). Accuracy of predicting axillary lymph node positivity by physical examination, mammography, ultrasonography, and magnetic resonance imaging. *Annals of Surgical Oncology*.

[33] Kim S. G., Friedman K., Patel S., Hagiwara M. (2016). Potential role of PET/MRI for imaging metastatic lymph nodes in head and neck cancer. *American Journal of Roentgenology*.

